# Knowledge, attitudes, and practices toward food allergies among early childhood educators in Taiwan: a cross-sectional study

**DOI:** 10.1186/s13223-026-01039-9

**Published:** 2026-05-05

**Authors:** Fang-Ting Lu, Chien-Che Ting, Kan-Hsuan Lin, Cheng-Han Lee, Shu-Hua Wang, Ping-Chen Wu, Shu-Yuan Zhou, Chien-Sheng Hsu, Jun-Kai Kao

**Affiliations:** 1 Frontier Molecular Medical Research Center in Children, Changhua Christian Children’s Hospital, 135 Nanhsiung Street, Changhua County, Changhua, 500 Taiwan; 2 Institute of Biomedical Sciences, National Chung Hsing University, Taichung city, Taiwan; 3 Doctoral Program in Tissue Engineering and Regenerative Medicine, National Chung Hsing University, Taichung City, Taiwan; 4 Department of Post-Baccalaureate Medicine, College of Medicine, National Chung Hsing University, Taichung city, Taiwan; 5 Department of Pediatric Gastroenterology, Hepatology and Nutrition, Changhua Christian Children’s Hospital, Changhua, Taiwan; 6 Research Division, Children’s Smile and Inspiration, Taichung city, Taiwan; 7 Department of Pediatrics, Changhua Christian Children’s Hospital, Changhua, Taiwan

**Keywords:** Anaphylaxis, Epinephrine autoinjector, Food allergy, Kindergarten staff, Knowledge, attitudes, practices, Taiwan

## Abstract

**Background:**

The prevalence of food allergies among Taiwanese children has risen to 10.4%, which is notably higher than that in many other Asian regions. However, despite this trend, Taiwan lacks specialized legislation or standardized national protocols for the management of food allergies in school settings. In this study, we assessed the knowledge, attitudes, and practices of kindergarten staff to identify key management gaps and unmet institutional needs.

**Methods:**

A cross-sectional, questionnaire-based study was conducted among 208 kindergarten staff members throughout Taiwan. Participants were categorized into major metropolitan areas (MMAs) and general urban-rural areas (GURAs) to evaluate regional disparities in resources and proficiency.

**Results:**

Although 88.0% of staff were aware of students with food allergies, there was a notable deficiency in clinical literacy. Only 13.5% recognized gluten as a trigger for severe reactions, and 82.2% were unaware of food-dependent exercise-induced anaphylaxis. Moreover, emergency preparedness was exceptionally low, with 74.0% of schools lacking formal response plans, 84.6% of staff unfamiliar with epinephrine autoinjectors, and only 2.9% of schools maintaining stocks of emergency medication. In addition, 53.4% of staff reported a refusal to administer life-saving medication due to legal anxiety. Although staff in MMAs reported higher clinical vigilance, experience, and a proactive willingness to perform on-site injections, a “uniform lack of competence” regarding emergency resources in both assessed regions. However, 83.6% of respondents expressed a strong willingness to pursue further training.

**Conclusions:**

The findings of this study reveal a systemic national failure in the food allergy safety policies of schools, rather than regional socioeconomic disparities. The discrepancy between high staff motivation and low clinical proficiency highlights the urgent need for mandatory, standardized professional development and hands-on emergency drills to ensure a safe educational environment.

## Introduction

Food allergies constitute a persistent health concern, characterized by a specific immune response following exposure to certain foods. In recent years, these allergies have emerged as a significant global public health priority, affecting both pediatric and adult populations. Worldwide, the prevalence of food allergies is estimated to range from approximately 1% to 10% [1, 2]. Allergic reactions can vary in severity and affect multiple organ systems, including those with cutaneous, respiratory, mucous, cardiovascular, and gastrointestinal effects, and in the most severe cases, these reactions can progress to life-threatening systemic anaphylaxis.

Compared with that in many other Asian regions, there is a significantly higher prevalence of food allergies in Taiwan. The findings of epidemiological studies have revealed that among Taiwanese children aged 6–7 years of age, the prevalence increased from 7.7% in 2004 to 10.4% in 2017, whereas that of adults reached 12.5% during the same period [3]. Comparatively, the childhood prevalence in the United States has recently stabilized at between 7.6% and 8.0% [2, 4], whereas surveys conducted in China, India, and Russia have reported considerably lower childhood prevalences ranging from 0.14% to 1.5% [5]. Similar to Taiwan, Japan has experienced a marked increase in the prevalence of food-related allergies among public elementary and middle school students, rising from 2.6% in 2004 to 6.3% in 2013, with current estimates indicating that these allergies affect approximately 8% of the Japanese pediatric population. According to the Ministry of Health and Welfare, prevalent food allergens in Taiwan encompass those to crustaceans, mangoes, peanuts, milk, eggs, tree nuts, sesame, gluten-containing cereals, soybeans, fish, and their derivatives [6]. In response to these trends, to ensure student safety, Japan has implemented stringent protocols, such as *the 2015 Guidelines for Food Allergy Management in School Lunches*, which delineate comprehensive catering procedures that contribute to prevent accidental exposure [7]. In contrast, Taiwan currently lacks specialized legislation or standardized national protocols specifically addressing food allergy management in school meal services.

In addition to the physical health risks, food allergies can have a pronounced influence on the psychosocial well-being of both patients and their caregivers. Research indicates that children with these allergies are more susceptible to anxiety [8], and given that students spend a significant proportion of their time in educational settings, reports indicate that 16–18% of food-allergic children experience allergic reactions within the school environment [9, 10]. This increasing prevalence highlights the urgent necessity for schools to provide comprehensive care, which includes safe meal preparation, preventive measures, and effective emergency management of allergic reactions.

International research examining the knowledge, attitudes, and practices (KAP) of kindergarten staff regarding food allergies among multiple regions worldwide, including Malaysia, Kuwait, Portugal, and Japan, have revealed that educators generally have low to moderate levels of proficiency in this regard. For example, a study conducted in Malaysia has reported that 86.3% of participants have insufficient overall knowledge [11], whereas in Kuwait, a mere 9.9% of staff expressed confidence in utilizing an epinephrine autoinjector [12], and in Portugal, only 11.7% felt assured in managing anaphylaxis [13]. Globally, significant challenges include low confidence in emergency response situations, the stigmatization of affected children, and a lack of formal training. In addition, there tend to be marked inconsistencies in implementation of emergency response plans in schools, with rates ranging from 25.2% to 86.3% [12, 14]. Moreover, a recent study in Miyagi Prefecture, Japan, has highlighted that amid a rising number of food allergy cases and the diversification of causative allergens, faculty members are experiencing considerable psychological burdens in striving to maintain a “zero-error” environment [15]. This burden is exacerbated in Taiwan, where Article 17 of the *Early Childhood Education and Care Act* stipulates that full-time nurses are required only in kindergartens with more than 200 students [16]. As a result, the responsibility for managing life-threatening emergencies predominantly falls on teaching staff, who must do so without the support of on-site medical professionals.

In Taiwan, ‘kindergarten’ is officially classified as ‘preschool’ under the Early Childhood Education and Care Act, serving children aged 2–6 years [16]. Presently, there is currently a notable deficiency in comprehensive research regarding the management of food allergies in kindergarten environments, the preparedness for emergency responses, and the specific challenges encountered by frontline staff. Against this backdrop, our objective in this cross-sectional study was to evaluate the knowledge, attitudes, and beliefs of Taiwanese kindergarten staff with respect to food allergies and to investigate the unmet needs of school personnel in managing these conditions.

## Materials and methods

### Study design and participant recruitment

In this cross-sectional, anonymous questionnaire-based study, we assessed the knowledge, attitudes, and practices (KAP) of kindergarten staff regarding food allergies. The study population comprised teaching and administrative personnel employed in both public and private kindergartens throughout Taiwan. Participant recruitment was conducted based on a hybrid approach, incorporating both physical outreach and digital distribution. Research information sheets were dispatched to kindergartens in different regions, including Taipei, New Taipei, Hsinchu, Taichung, Changhua, Nantou, Yunlin, and Kaohsiung. Concurrently, to ensure comprehensive coverage of the target population, the survey was made accessible via online links and QR codes, which were disseminated via professional faculty networks and specialized kindergarten groups.

## Regional classification

To examine potential disparities in food allergy management in differing socioeconomic contexts, participants were divided into two distinct groups based on regional population density, economic infrastructure, and the availability of public resources. The major metropolitan area (MMA) group consisted of participants from highly urbanized centers, specifically Taipei, New Taipei, Taichung, and Kaohsiung, whereas the general urban–rural area (GURA) group encompassed all other surveyed regions, characterized by comparatively lower population densities and differing levels of infrastructure. This classification facilitated a comparative analysis of how regional urbanization levels influence the KAP of kindergarten staff.

## Instrumentation

The research instrument, titled the “Survey on Food Allergy Awareness among Kindergarten Staff,” consisted of a self-administered questionnaire comprising 39 items organized into five principal dimensions. The first dimension gathered demographic and background information, encompassing personal and institutional characteristics. The second evaluated experience in food allergy care, emphasizing the frequency of interactions with allergic students and the specific methods utilized to identify these individuals. The third assessed knowledge and clinical awareness, addressing the recognition of clinical manifestations, common allergens (such as gluten), diagnostic “gold standards” (e.g., the oral food challenge), and history of anaphylaxis training. The fourth, emergency management, examined awareness of anaphylaxis protocols, familiarity and proficiency with epinephrine autoinjectors (EpiPens), and the availability of institutional response equipment. Lastly, the survey examined challenges and stress assessment, measuring implementation-associated difficulties in school meal services and the psychological stress associated with managing different allergic conditions, including asthma, atopic dermatitis, and food allergies, quantified via a 11-point Likert scale (0–10). Prior to application, the instrument was reviewed by a panel of pediatric allergists to ensure content validity.

### Statistical analysis

Statistical analyses were conducted using IBM SPSS Statistics version 30.0, with the significance threshold established at *p* < 0.05. Descriptive statistics were employed to summarize the data, with frequencies (n) and percentages (%) used for categorical variables, whereas continuous variables are presented as the means ± standard deviations (SD) or standard errors (SEM). For comparative analysis, chi-square or Fisher’s exact tests were applied to evaluate associations between categorical variables between demographic groups, whereas differences between regions and staff categories were assessed using independent samples *t*-tests or a one-way analysis of variance (ANOVA), with repeated-measures ANOVA being employed to compare stress levels among different allergic conditions. To control for the inflation of Type I errors during multiple comparisons, the false discovery rate (FDR) method or Bonferroni post hoc tests were applied where appropriate.

## Ethical considerations

This study received approval from the Institutional Review Board (IRB No.:250524). Prior to completing the anonymous survey, all participants were informed of the study’s purpose and provided informed consent.

## Results

### Participant demographics and institutional background

From the 2000 information sheets initially distributed, we received 208 valid responses, representing a percentage response of approximately 10%. The majority of respondents were female (96.6%), with childcare provider (53.9%) and teachers (26.9%) constituting the primary professional categories. The staff were established to have substantial experience, with 73.1% having gained over 5 years of teaching experience. Geographically, participants were categorized into MMAs (35.1%) and GURAs (64.9%), with no significant differences observed between these regions regarding staff gender, job titles, or teaching tenure (Table 1).

**Table 1:** Participant demographics

Sex, female (%	MMA (%, *n* = 73)	GURA (%, *n* = 135)	Total (%, *N* = 208)	*p*-value
	94.4	97.8	96.6	0.230
Length of teaching tenure				
< 1 year	4.1	10.4	8.2	0.440
1–3 year	8.2	8.1	8.2	
3–5 year	12.3	9.6	10.6	
> 5 year	75.3	71.9	73.1	
Faculty and staff (select all that apply)				
Principal	8.2	5.9	6.7	0.275
Director	13.7	8.9	10.6	
Teacher	34.3	23.0	26.9	
Childcare provider	48.0	57.0	53.9	
Others	8.2	11.9	10.6	
Number of students in the class (mean ± SD)	20.98 ± 8.50	19.62 ± 7.09	20.08 ± 7.60	0.245

Unless otherwise noted, data are presented as percentages. Continuous variables (stress levels) are presented as mean ± SD. Statistical significance was determined using Chi-square test, Fisher’s exact test, or independent t-test where appropriate. Asterisks indicate a significant difference between regions (*p* < 0.05). MMA: Major Metropolitan Areas; GURA: General Urban-Rural Areas

## Food allergy care experience and identification methods

**Fig. 1:**
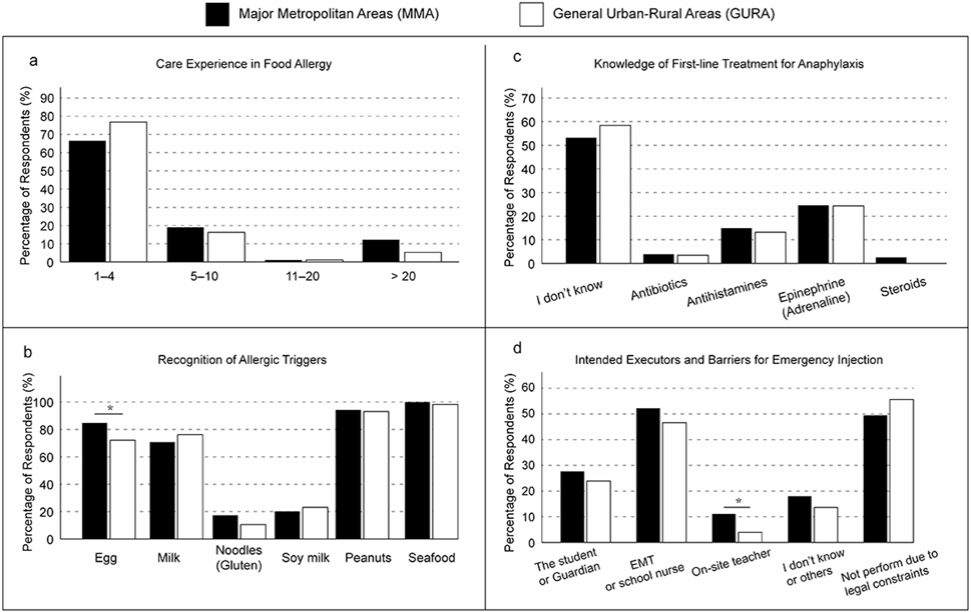
Experience, trigger identification, and emergency intervention regarding food allergy among preschool staff in MMA and GURA. **a** Distribution of care experience: percentage of staff based on the total number of students with food allergies they have taught throughout their careers. Most respondents were in the 1–4 students category, with no significant regional differences observed. **b** Identification of allergic triggers: proportion of staff correctly identifying specific potential triggers (multiple responses allowed). Knowledge regarding noodles (gluten) remained notably low across both groups. **c** First-line medication knowledge for anaphylaxis: the percentage of respondents selecting various treatments for life-threatening anaphylactic shock. Over 50% of staff in both regions indicated “I don’t know,” and a substantial proportion incorrectly identified antihistamines as the primary treatment instead of epinephrine (adrenaline). **d** Intended executors and legal barriers for emergency injection: proportion of staff identifying who would perform an EpiPen injection (multiple responses allowed). While EMT or school nurses were the most frequently selected intended executors, MMA staff were significantly more likely to identify on-site teachers as potential executors compared to GURA staff (*p* < 0.05). However, more than half of the respondents in both groups indicated that they would not perform the injection due to regulatory and legal constraints. Data are presented as percentages. Asterisks (*) indicate statistical significance at the *p* < 0.05 level based on Chi-square or Fisher’s exact tests. MMA: Major Metropolitan Areas; GURA: General Urban-Rural Areas

Although 88.0% of staff were cognizant of students with food allergies within their schools, the actual experience in providing direct care was relatively limited. Approximately 73.5% of respondents reported having encountered fewer than five students with allergies throughout their professional careers (Fig. 1a). Notably, staff in MMAs (31.5%) were significantly more likely to have managed students experiencing severe allergic reactions compared with those in GURAs (18.5%) (*p* = 0.034). In terms of identification, although the majority of staff relied on parental notification (95.7%) or school forms (55.8%), a concerning 33.7% of teachers identified allergies based om their observations if students’ physical reactions following consumption (Table 2).

**Table 2:** Food allergy care experience and identification protocols in kindergartens

	MMA (%, *n* = 73)	GURA (%, *n* = 135)	Total (%, *N* = 208)	*p*-value
History of caring for students with severe food allergies	31.5	18.5	23.1	0.034*
Have identification systems for students with food allergies	53.4	52.6	52.9	0.910
Awareness of students with food allergies in the current school/class	91.8	85.9	88.0	0.220
Number of students with food allergies in the current class				
0	30.1	46.7	40.9	0.051
1–4	65.8	52.6	57.2	
5–10	2.7	0.7	1.4	
10–20	1.4	0.0	0.5	
Students excluding specific foods based on blood test results (IgE)	43.8	40.7	41.8	0.670
Methods to identify students with food allergies (Select all that apply)				
Questionnaire	56.2	55.6	55.8	0.920
Medical certificates	15.1	8.2	10.6	
Parental/guardian notification	93.2	97.0	95.7	
Blood test results	9.6	5.2	6.7	
Observation of symptoms post-ingestion	35.6	32.6	33.7	
Others	0.0	1.5	1.0	

Unless otherwise noted, data are presented as percentages. Statistical significance was determined using the Chi-square test or Fisher’s exact test where appropriate. Asterisks indicate a significant difference between regions (*p* < 0.05). MMA: Major Metropolitan Areas; GURA: General Urban-Rural Areas

### Clinical knowledge and training gaps

Significant deficiencies in food allergy knowledge were identified across multiple dimensions. Although more than 90% of staff reported examining food labels, only 13.5% accurately identified gluten (noodles) as a potential trigger for severe allergic reactions (Fig. 1b). Diagnostic literacy was also found to be inadequate, with 63.9% of staff being found to be unaware that the oral food challenge is considered the “gold standard” for diagnosis, and 41.8% were exclusively dependent on blood test results (IgE) to justify food avoidance (Table 2). In addition, 82.2% of staff were uninformed with respect to the nature and severity of food-dependent exercise-induced anaphylaxis. Overall, the survey results indicated that formal training appears to be insufficient, as only 13.9% of participants had ever received instruction in food allergy or anaphylaxis management (Table 3).

**Table 3:** Proficiency in food allergy recognition and emergency response protocols

	MMA (%, *n* = 73)	GURA (%, *n* = 135)	Total (%, *N* = 208)	*p*-value
Self-reported familiarity with clinical symptoms of food allergies				
Unfamiliar to slightly familiar	49.3	61.5	57.2	0.045*
Moderately familiar	30.1	29.6	29.8	
Very to extremely familiar	20.5	8.9	13.0	
Awareness of exercise as a co-factor in triggering food allergy symptoms (FDEIA)	20.5	16.3	17.8	0.440
Awareness of the oral food challenge (OFC) as the gold standard for FA diagnosis	32.9	37.8	36.1	0.482
Estimated prevalence of food allergies among 6–7-year-old children in Taiwan				
< 3%	5.5	8.9	7.7	0.190
3–6%	31.5	38.5	36.1	
6–9%	30.1	32.6	31.7	
Over 10%	32.9	20.0	24.5	
Previous participation in training for anaphylaxis management	15.1	13.3	13.9	0.730
Have never heard of EpiPen (epinephrine auto-injectors)	84.9	84.4	84.6	0.930
Proficiency in administering an EpiPen	6.8	5.2	5.8	0.620
Existence of a school-wide food allergy emergency response plan	31.5	23.0	26.0	0.180
Availability of emergency allergy medications in school stock	5.5	1.5	2.9	0.100

Unless otherwise noted, data are presented as percentages. Statistical significance was determined using the Chi-square test or Fisher’s exact test where appropriate. Asterisks indicate a significant difference between regions (*p* < 0.05). MMA: Major Metropolitan Areas; GURA: General Urban-Rural Areas

### Emergency management and medication awareness

The survey also revealed notable deficiencies regarding the institutional readiness for managing life-threatening reactions, as evidenced by the 74.0% of respondents indicating that their schools lack a formal emergency response plan (Table 3). Knowledge of first-line treatments is similarly inadequate, with only 24.5% of staff correctly identifying epinephrine as the primary medication for fatal anaphylaxis (Fig. 1c). With regards to EpiPens, 84.6% of teachers were unfamiliar with the device, and 94.2% were unaware of the correct administration procedure. Moreover, a mere 2.9% of schools covered by the survey were found to maintain a stock of emergency allergy medications (Table 3).

### Psychological stress, legal concerns, and motivation

Managing allergic diseases presents a quantifiable psychological burden on educational staff. Compared with atopic dermatitis (3.33 ± 2.62) or food allergies (2.96 ± 2.75), teachers reported significantly elevated levels of stress associated with the management of asthma (4.16 ± 3.16) (Repeat ANOVA, *p* < 0.017) (Table 4). Notably, it emerged that a primary impediment to emergency care is legal anxiety, with 53.4% of teachers indicating that they would decline to administer an EpiPen due to perceived regulatory and legal liabilities (Fig. 1d). Despite these challenges, there is, nevertheless, a strong professional impetus for reform, with 83.6% of staff expressing a willingness to pursue further training in the management of childhood food allergies.

**Table 4:** Staff perspectives on the psychosocial burden of students and associated occupational stress

	MMA (%, *n* = 73)	GURA (%, *n* = 135)	Total (%, *N* = 208)	*p*-value
Perceived impact of food allergies on students’ daily lives				
No impact observed	42.5	51.9	48.6	0.370
Impaired social interactions	2.7	0.74	1.4	
Emotional and psychological distress	39.7	28.9	32.7	
Interruption of learning activities	8.2	11.1	10.1	
Others	6.9	7.4	7.2	
Challenges encountered in school meal services and FA management (Select all that apply)				
Complexity of food selection or elimination	43.8	42.2	42.8	0.877
Rising prevalence of students with food allergies	23.3	16.3	18.8	
Insufficient collaboration among teachers, guardians, and physicians	5.5	7.4	6.7	
Inadequate manpower and specialized equipment	5.5	5.9	5.8	
No significant challenges encountered	50.7	50.4	50.5	
Others	2.7	4.4	3.9	
Perceived stress level regarding students with food allergies (mean ± SD)	3.42 ± 2.91	2.71 ± 2.63	2.96 ± 2.75	0.073
Perceived stress level regarding students with atopic dermatitis (mean ± SD)	3.29 ± 2.76	3.36 ± 2.55	3.33 ± 2.61	0.860
Perceived stress level regarding students with asthma (mean ± SD)	4.40 ± 3.27	4.03 ± 3.10	4.16 ± 3.16	0.420

Unless otherwise noted, data are presented as percentages. Continuous variables (stress levels) are presented as mean ± SD. Statistical significance was determined using Chi-square test, Fisher’s exact test, or independent t-test where appropriate. Asterisks indicate a significant difference between regions (*p* < 0.05). MMA: Major Metropolitan Areas; GURA: General Urban-Rural Areas

### Regional disparities: MMA versus GURA

The survey data indicated that throughout Taiwan, there is a “uniform lack of competence,” although there tends to be minor regional differences in this regard. Staff in MMAs reported greater self-confidence in recognizing clinical symptoms (20.5% vs. 8.9%, *p* = 0.045) (Table 3) and noted a more pronounced influence of allergies on the emotional and psychological distress of students (39.7% vs. 28.9%) (Table 4). A significant geographical variation was observed regarding emergency response preferences, with MMA staff demonstrating a markedly higher propensity to administer on-site injections (11.0%) compared to their counterparts in GURA (3.7%) (*p* = 0.039) (Fig. 1d).

## Discussion

### Discrepancy between prevalence and practical experience

Despite clinical data indicating a prevalence of 10.4% in Taiwan, nearly 60% of staff reported having very limited practical experience with respect the management of food allergies. Previous prevalence-associated studies in Taiwan have been based predominantly on the findings of parent-reported questionnaires rather than clinical diagnoses. It can thus be speculated that this discrepancy might reflect a significant under-reporting by parents due to fear, stigma, or a cognitive gap within school settings [17, 18]. 

### Gaps in clinical literacy and underestimation of psychosocial impacts

Our findings also indicate a notable disparity among kindergarten staff regarding general awareness and clinical proficiency. Although 88.0% of participants were cognizant of students with food allergies in their institutions, there was a clear lack of specific medical knowledge. Only 13.5% accurately identified gluten as a major allergen, and awareness of the gold standard diagnostic tool, the oral food challenge, was minimal. This observation is consistent with the findings of international studies, which have indicated that whereas educators often express high levels of concern regarding food allergies, they frequently lack the medical literacy necessary for safe management [19–21], with more than one-third of staff identifying allergies based on “observation after consumption.” In the absence of adequate clinical knowledge, a dependence on observations substantially heightens the risk of delayed intervention and potentially fatal outcomes during life-threatening reactions [21]. Of particular concern in this regard is that most teachers failed to recognize food-dependent exercise-induced anaphylaxis [22], a condition in which shock is triggered only by the combination of consuming a specific food and exercising. As staff may inadvertently place allergic children in danger by scheduling physical activities shortly after meals, this accordingly poses a hidden risk. Beyond clinical risks, there remains a prominent lack of perception regarding the psychosocial well-being of students. Despite international literature highlighting the fact that children with food allergies frequently encounter bullying, ridicule, harassment, and denial of their allergies [23, 24], nearly half of respondents were under the impression that food allergies had no substantial impact on students. The Taiwanese early childhood education sector thus appears to underestimate the psychosocial challenges faced by children with allergies (Table 4).

### The visibility bias of perceived stress

Currently, there is a paucity of comparative evidence regarding stress among teacher with respect to different allergic diseases. Our findings indicate that compared with food allergies, staff experience significantly greater stress when managing asthma (*p* < 0.017). Nonetheless, given their increasing prevalence, diverse triggers, dietary uncertainties, and inadequate collaboration among teachers, guardians, and physicians, the management of food allergies can impose considerable strain [15, 23]. Our findings indicate that school staff tend to prioritize conditions with overt clinical symptoms, such as asthma, and, consequently, may overlook the rarer, albeit life-threatening, risk of food allergy-induced anaphylaxis.

### Deficiencies in emergency response and EpiPen administration

Globally, between 40% and 80% of educators lack formal training regarding the management of food allergies or anaphylaxis [13, 25, 26]. In Taiwan, this deficiency is generally more pronounced, with only 13.9% of kindergarten staff trained in managing anaphylactic incidents. Notably, over 80% of the staff are unfamiliar with the use of EpiPens, and a mere 2.9% of schools maintain stocks of emergency medication, indicating that Taiwan’s early childhood education system is inadequately prepared to deal appropriately with potentially fatal allergic reactions. Similar deficiencies in institutional preparedness have been documented in other Asian contexts, such as in South Korea [26]. Such institutional vulnerabilities in Taiwan are largely rooted in the current regulatory framework. Specifically, Article 11 of the *Early Childhood Education and Care Act* restricts medication assistance to drugs prescribed to individual students, effectively preventing schools from maintaining a “stock” of epinephrine [16]. Preschool teachers in Taiwan face critical legal dilemmas. According to Article 294 of the *Criminal Code*, they risk prosecution for “offenses of abandonment” if they fail to provide necessary protection during a student’s anaphylactic shock [27]. Conversely, 53.4% of surveyed educators expressed reluctance to administer EpiPens due to conflicting legal concerns. Although the *Emergency Medical Care Act* offering immunity to non-medical rescuers [28, 29], Article 28 of the *Physicians Act* still categorizes injections by unlicensed individuals as “illegal medical practice,” which is punishable by up to five years of imprisonment [30]. This situation highlights the current impasse, where, despite the generally professed willingness to assist, there is prevailing inaction [23, 25]. To address this reluctance and enhance self-efficacy, school training must shift toward practical drills [31, 32]. More importantly, this underscores the need for advocacy-based organizations to reconcile discrepancies between medical guidelines and legislation. However, Taiwan currently lacks such non-profit organizations.

### A national systemic failure

With respect to food allergies, research has provided clear evidence of a global “urban–rural divide.” Notably, it has been established that there is a significantly higher prevalence of food allergies among children growing up in urban environments, and urban schools, which accordingly face greater challenges, are generally more well prepared to address these challenges [2, 21, 25, 33, 34]. However, our findings indicate that although teachers in MMAs generally have heightened clinical vigilance, experience, and a proactive willingness to perform on-site injections, there is a consistent lack of emergency training and an absence of institutionalized emergency action plans and EpiPen availability among both MMAs and GURAs in Taiwan. These findings thus provide evidence to indicate that rather than constituting a regional socioeconomic issue, the mismanagement of food allergies in Taiwanese kindergartens represents a systemic national failure in school safety policy.

### High motivation as a catalyst for change

Empirical evidence indicates that formal or simulation-based training can substantially contribute to enhancing both preparedness and attitude [19–21, 32]. The most promising finding of the present study is that 83.6% of Taiwanese educators profess a strong willingness to further their understanding of childhood food allergies. This high level of motivation among frontline personnel accordingly establishes a solid foundation for the implementation of mandatory school safety policies and standardized professional development programs.

### Strengths and Limitations

To the best of our knowledge, this study represents the first comparative analysis of food allergy management among kindergarten staff in the urban and rural areas of Taiwan, emphasizing the influence of legal anxiety and psychological stress. However, despite these valuable insights, to a certain extent, the limited sample size and cross-sectional design constrain the generalizability of the results. Future research should thus incorporate qualitative methodologies to investigate gaps in the communication between parents and teachers, thereby enhancing the efficacy of collaborative care models [34]. 

## Data Availability

The datasets used and/or analyzed during the current study are available from the corresponding author on reasonable request.
